# High-pressure synthetic (Na_0.97_Mg_0.03_)(Mg_0.43_Fe_0.17_^3+^Si_0.40_)Si_2_O_6_, with six-coordinated silicon, isostructural with *P*2/*n* omphacite

**DOI:** 10.1107/S1600536812002966

**Published:** 2012-01-31

**Authors:** Esther S. Posner, Jürgen Konzett, Daniel J. Frost, Robert T. Downs, Hexiong Yang

**Affiliations:** a Department of Geosciences, University of Arizona, 1040 E. 4th Street, Tucson, AZ 85721-0077, USA; b Institut für Mineralogie und Petrographie, Universität Innsbruck, Innsbruck, Austria; c Bayerisches Geoinstitut, Universität Bayreuth, Bayreuth, Germany

## Abstract

The title compound, (sodium magnesium) [magnesium iron(III) silicon] disilicate, (Na_0.97_Mg_0.03_)(Mg_0.43_Fe_0.17_^3+^Si_0.40_)Si_2_O_6_, is isotypic with ordered *P*2/*n* omphacite. Its structure is characterized by single chains of corner-sharing SiO_4_ tetra­hedra, extending along the *c* axis, which are crosslinked by bands of edge-sharing octa­hedra (site symmetry 2), statistically occupied by (Mg^2+^ + Fe^3+^ + Si^4+^). Between the bands built up of the octahedra are two non-equivalent highly distorted six-coordinated sites (site symmetry 2), statistically occupied by (Na + Mg). In contrast to omphacites, the great differences in size and charge between Mg^2+^ and Si^4+^ result in complete, rather than partial, ordering of Mg and Si into two distinct octa­hedral sites, whereas Fe^3+^ is disordered between the two sites. The octa­hedron filled by (Mg + Fe) is larger and markedly more distorted than that occupied by (Si + Fe). The average (Mg + Fe)—O and (^VI^Si + Fe)—O bond lengths are 2.075 and 1.850 Å, respectively.

## Related literature

For structures of high-pressure synthetic clinopyroxenes with six-coordinated Si, see: Angel *et al.* (1988 ▶); Yang & Konzett (2005 ▶); Yang *et al.* (2009 ▶). For structures of ordered *P*2/*n* omphacites, see: Curtis *et al.* (1975 ▶); Matsumoto *et al.* (1975 ▶); Rossi *et al.* (1983 ▶). For background on the stability of clino­pyroxenes at high pressures and temperatures, see: Gasparik (1989 ▶); Konzett *et al.* (2005 ▶). For general background on materials with six-coordinated silicon, see: Finger & Hazen (1991 ▶). For the geologic occurrence of clinopyroxene with six-coordinated Si, see: Wang & Sueno (1996 ▶). For spectroscopic measurements on *P*2/*n* clinopyroxenes, see: Boffa Ballaran *et al.* (1998 ▶); Yang *et al.* (2009 ▶). For general information on polyhedral distortion and ionic radii, see: Robinson *et al.* (1971 ▶) and Shannon (1976 ▶), respectively.

## Experimental

### 

#### Crystal data

(Na_0.97_Mg_0.03_)(Mg_0.43_Fe_0.17_Si_0.40_)Si_2_O_6_
*M* *_r_* = 206.39
Monoclinic, 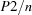

*a* = 9.4432 (8) Å
*b* = 8.6457 (7) Å
*c* = 5.2540 (5) Å
β = 108.003 (6)°
*V* = 407.95 (6) Å^3^
*Z* = 4
Mo *K*α radiation
μ = 1.69 mm^−1^
*T* = 293 K
0.06 × 0.05 × 0.05 mm

#### Data collection

Bruker APEXII CCD area-detector diffractometer
Absorption correction: multi-scan (*SADABS*; Sheldrick 2005 ▶) *T*_min_ = 0.906, *T*_max_ = 0.920
6586 measured reflections
1481 independent reflections
980 reflections with *I* > 2σ(*I*)
*R*_int_ = 0.033

#### Refinement

*R*[*F* ^2^ > 2σ(*F* ^2^)] = 0.036
*wR*(*F* ^2^) = 0.085
*S* = 1.07
1481 reflections
96 parameters
3 restraints
Δρ_max_ = 0.51 e Å^−3^
Δρ_min_ = −0.66 e Å^−3^

### 

Data collection: *APEX2* (Bruker, 2004 ▶); cell refinement: *SAINT* (Bruker, 2004 ▶); data reduction: *SAINT*; program(s) used to solve structure: *SHELXS97* (Sheldrick, 2008 ▶); program(s) used to refine structure: *SHELXL97* (Sheldrick, 2008 ▶); molecular graphics: *XtalDraw* (Downs & Hall-Wallace, 2003 ▶); software used to prepare material for publication: *publCIF* (Westrip, 2010 ▶).

## Supplementary Material

Crystal structure: contains datablock(s) I, global. DOI: 10.1107/S1600536812002966/fj2504sup1.cif

Structure factors: contains datablock(s) I. DOI: 10.1107/S1600536812002966/fj2504Isup2.hkl

Additional supplementary materials:  crystallographic information; 3D view; checkCIF report

## Figures and Tables

**Fig. 1.**
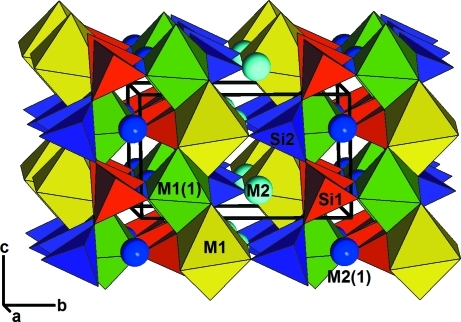
Crystal structure of P2/n (Na0.97Mg0.03)(Mg0.43Fe0.17Si0.40)Si2O6 clinopyroxene.

**Fig. 2.**
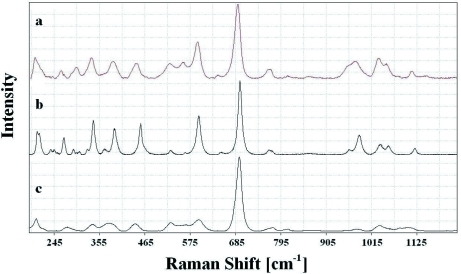
Raman spectra of clinopyroxenes. (a) P2/n (Na0.97Mg0.03)(Mg0.43Fe0.17Si0.40)Si2O6 (this study), (b) P2/n Na(Mg0.45Al0.10Si0.45)Si2O6 (Yang et al., 2009) and (c) C2/c (Na0.97Mg0.03)(Mg0.37Al0.30Si0.33)Si2O6 (Yang et al., 2009).

## supplementary crystallographic information

### Comment

The coordination of silicon with oxygen in crystalline materials is crucial for our understanding of the structure and composition of the Earth's interior because together they account for 63% of the atoms in the planet. In general, silicon is four-coordinated (^IV^Si) in the Earth's crust and upper mantle, but six-coordinated (^VI^Si) in the lower mantle (*e.g.*, Finger & Hazen, 1991). In the Earth's transition zone (between depths of 410 and 670 km), minerals are found to contain both ^IV^Si and ^VI^Si. Phase transitions that involve a change in the Si coordination may affect many important physical and chemical properties of materials, such as density, bulk moduli, and elasticity, which, when coupled with seismic observations, can provide vital information on the complex constituents of the Earth's mantle.

Clinopyroxenes, one of the major rock-forming minerals of the Earth's upper mantle, were long assumed to contain ^IV^Si only. Studies of pyroxenes synthesized at high temperatures and pressures, however, have revealed their capacity to accommodate both ^IV^Si and ^VI^Si (Angel *et al.* 1988; Konzett *et al.* 2005; Yang & Konzett 2005; Yang *et al.*, 2009), pointing to their possible stabilities at higher pressures. In particular, Angel *et al.* (1988) reported a high-pressure Na(Mg_0.5_Si_0.5_)Si_2_O_6_ clinopyroxene (designated as NaPx hereafter), which is isostructural with ordered *P*2/*n* omphacite, with ^VI^Si and Mg fully ordered into two distinct octahedral sites. Later studies of NaPx-CaMgSi_2_O_6_ (diopside) and NaPx-NaAlSi_2_O_6_ (jadeite) solid solutions uncovered a symmetry transition from an ordered *P*2/*n* to a disordered *C*2/*c* structure as ^VI^Si content decreases (Yang & Konzett, 2005; Yang *et al.*, 2009). The natural occurrence of a clinopyroxene containing ^VI^Si, (Na_0.16_Mg_0.84_)(Mg_0.92_Si_0.08_)Si_2_O_6_, was reported by Wang & Sueno (1996) as an inclusion in a diamond from a kimberlite in China. According to the phase stability relations for the NaPx-Mg_2_Si_2_O_6_ (enstatite) join (Gasparik, 1989), this inclusion crystallized at pressures greater than 16.5 GPa, or at a depth within the Earth's transition zone (~500 km). The foremost implications of this finding include that (1) some portions of the Earth's upper mantle may contain a greater ratio of Na/Al than previously inferred from the xenolith chemistry, and (2) clinopyroxenes may be one of potential candidates as a silica-rich phase in the Earth's mantle. To gain more insights into the systematics on the crystal chemistry and stability field of pyroxenes with ^VI^Si, we conducted a structure refinement of a high-pressure synthetic NaPx-NaFeSi_2_O_6_ (aegirine) solid solution (designated as NaPxFe hereafter) based on the single-crystal X-ray diffraction data.

NaPxFe is isotyic with *P*2/*n* omphacite (Matsumoto *et al.* 1975; Curtis *et al.* 1975; Rossi *et al.* 1983) and NaPx (Angel *et al.*, 1988; Yang *et al.*, 2009). Its structure is characterized by a distorted closest-packed array of oxygen atoms with a layer of single silicate chains, extending along *c*, formed by Si1O_4_ and Si2O_4_ tetrahedra, alternating with a layer containing two distinct, edge-sharing octahedra M1 and M1(1), occupied by (Mg^2+^ + Fe^3+^ + Si^4+^). Also in the octahedral layer are two nonequivalent, considerably distorted six-coordinated sites M2 and M2(1), occupied by (Na + Mg) (Fig. 1). Note that Na in clinopyroxenes has been regarded to be eight-coordinated in all previous studies. However, our electron-density and bond-valence sum calculations indicate that Na in NaPxFe is actually six-coordinated. In contrast to omphacites, the great differences in size and charge between Mg^2+^ and Si^4+^ result in complete, rather than partial, ordering of Mg at M1 and Si at M1(1), while Fe is disordered between the two sites. An inspection of structure data for pyroxenes containing ^VI^Si shows that, as the respective contents of Mg and Si in the M1 and M1(1) sites decreases from NaPx to Na(Mg_0.45_Al_0.10_Si_0.45_)Si_2_O_6_ (Angel *et al.*, 1988; Yang *et al.*, 2009) and NaPxFe, the average M1—O bond length decreases from 2.092 to 2.084 and 2.075 Å, respectively, whereas the average M1(1)-O bond distance increases from 1.807 Å to 1.813 and 1.850 Å, respectively. This observation is evidently a direct consequence of the differences in ionic radii among Mg^2+^ (0.72 Å), Fe^3+^ (0.645 Å), Al^3+^ (0.535 Å), and ^VI^Si^4+^ (0.40 Å) (Shannon, 1976). The coupled changes in the average M1—O and M1(1)-O bond distances from NaPx to Na(Mg_0.45_Al_0.10_Si_0.45_)Si_2_O_6_ and NaPxFe lead to a substantial reduction in the size mismatch between the two edge-shared octahedra, as well as the degree of distortion of the M1 octahedron in terms of the octahedral angle variance (OAV) and octahedral quadratic elongation (OQE) (Robinson *et al.*, 1971). The OAV and OQE values are 107.2 and 1.035, respectively, for the M1 octahedron in NaPx, 101.4 and 1.033 in Na(Mg_0.45_Al_0.10_Si_0.45_)Si_2_O_6_, and 88.2 and 1.029 in NaPxFe. Interestingly, as the geometric differences between the M1 and M1(1) octahedra decreases with decreasing ^VI^Si content from NaPx to Na(Mg_0.45_Al_0.10_Si_0.45_)Si_2_O_6_ and NaPxFe, the difference between the mean M2—O and M2(1)-O bond distances is also reduced, which is 0.123, 0.113, and 0.096 Å for NaPx, Na(Mg_0.45_Al_0.10_Si_0.45_)Si_2_O_6_, and NaPxFe, respectively. Conceivably, as the ^VI^Si content is further decreased, the *P*2/*n* structure will eventually transform to the *C*2/*c* structure, in which M1 and M1(1) become identical, so do M2 and M2(1), and Si1 and Si2.

Plotted in Figure 2 are Raman spectra of NaPxFe and two clinopyroxenes in the NaPx-jadeite system for comparison, one with *P*2/*n* symmetry and the other *C*2/*c* (Yang *et al.*, 2009). The detailed assignment of Raman bands for clinopyroxenes containing ^VI^Si has been discussed by Yang *et al.* (2009). Generally, the Raman spectra of pyroxenes can be classified into four regions. Region 1 includes the bands between 800 and 1200 cm^-1^, which are assigned as the Si—O stretching vibrations in the SiO_4_ tetrahedra. Region 2 is between 630–800 cm^-1^, which includes the bands attributable to the Si—O—Si vibrations within the silicate chains. Region 3, from 400 to 630 cm^-1^, includes the bands that are mainly associated with the O—Si—O bending modes of SiO_4_ tetrahedra. The bands in Region 4, which spans from 50 to 400 cm^-1^, are of a complex nature, chiefly due to lattice vibration modes, polyhedral librations and M—O interactions, as well as possible O—Si—O bending. As noted by Boffa Ballaran *et al.* (1998) and Yang *et al.* (2009), the *C*2/*c*-to-*P*2/*n* transformation is characterized by the splitting of many observable Raman bands in the *C*2/*c* structure into doublets in the *P*2/*n* structure, consistent with the doubled number of independent atomic sites in the *P*2/*n* structure relative to that in the *C*2/*c* structure. Compared to the Raman spectrum of *P*2/*n* Na(Mg_0.45_Al_0.10_Si_0.45_)Si_2_O_6_ (Sample J2, Yang *et al.*, 2009), most Raman bands for NaPxFe are noticeably broader, indicating the increased positional disorder of atoms in our sample, which further suggests that the chemistry of our sample is closer to the *P*2/*n*-to-*C*2/*c* transition than that of Na(Mg_0.45_Al_0.10_Si_0.45_)Si_2_O_6_ (Yang *et al.*, 2009), in agreement with the conclusion we derived above from the structure refinement.

### Experimental

The specimen used in this study was synthesized in a multi-anvil apparatus at 15 GPa and 1500 °C for 23.2 h (run #JKB2006–12) and then rapidly quenched (< 5 s) to ambient conditions. The crystal chemistry was determined with a Jeol electron microprobe on the same single-crystal used for the X-ray intensity data collection. The average composition of nine analysis points yielded a chemical formula (normalized on the basis of 6 oxygen atoms and 4 cations while maintaining charge-balance) (Na_0.97_Mg_0.03_)(Mg_0.43_Fe_0.17_Si_0.40_)Si_2_O_6._

The Raman spectrum of (Na_0.97_Mg_0.03_)(Mg_0.43_Fe_0.17_Si_0.40_)Si_2_O_6_ was collected from a randomly oriented crystal at 100% power on a Thermo Almega microRaman system, using a 532 nm solid-state laser, and a thermoelectrically cooled CCD detector. The laser is partially polarized with 4 cm^-1^ resolution and a spot size of 1 µm.

### Refinement

Throughout the structure refinements, the chemical composition of the crystal was fixed to that determined from electron microprobe analysis. From crystal-chemical considerations, all Mg and ^VI^Si were assigned to the M1 and M1(1) sites, respectively, with the rest filled by Fe^3+^ for both sites. Because the average distance of M2—O is shorter than that of M2(1)-O, we assigned 0.03 Mg apfu to the M2 site. The highest residual peak in the difference Fourier maps was located at (0.7991, 0.0789, 0.2448), 0.54 Å from M2, and the deepest hole at (0.2602, 0.1856, 0.1853), 0.48 Å from M1(1).

### Crystal data

**Table:**

(Na_0.97_Mg_0.03_)(Mg_0.43_Fe_0.17_Si_0.40_)Si_2_O_6_	*F*(000) = 409
*M**_r_* = 206.39	*D*_x_ = 3.360 Mg m^−^^3^
Monoclinic, *P*2/*n*	Mo *K*α radiation, λ = 0.71073 Å
Hall symbol: -P 2yac	Cell parameters from 1071 reflections
*a* = 9.4432 (8) Å	θ = 3.3–35.6°
*b* = 8.6457 (7) Å	µ = 1.69 mm^−^^1^
*c* = 5.2540 (5) Å	*T* = 293 K
β = 108.003 (6)°	Cube, pale gray
*V* = 407.95 (6) Å^3^	0.06 × 0.05 × 0.05 mm
*Z* = 4	

### Data collection

**Table:**

Bruker APEXII CCD area-detector diffractometer	1481 independent reflections
Radiation source: fine-focus sealed tube	980 reflections with *I* > 2σ(*I*)
graphite	*R*_int_ = 0.033
φ and ω scan	θ_max_ = 32.5°, θ_min_ = 3.3°
Absorption correction: multi-scan (*SADABS*; Sheldrick 2005)	*h* = −14→14
*T*_min_ = 0.906, *T*_max_ = 0.920	*k* = −13→10
6586 measured reflections	*l* = −7→7

### Refinement

**Table:**

Refinement on *F*^2^	Primary atom site location: structure-invariant direct methods
Least-squares matrix: full	Secondary atom site location: difference Fourier map
*R*[*F*^2^ > 2σ(*F*^2^)] = 0.036	*w* = 1/[σ^2^(*F*_o_^2^) + (0.0264*P*)^2^ + 0.6629*P*] where *P* = (*F*_o_^2^ + 2*F*_c_^2^)/3
*wR*(*F*^2^) = 0.085	(Δ/σ)_max_ = 0.001
*S* = 1.07	Δρ_max_ = 0.51 e Å^−^^3^
1481 reflections	Δρ_min_ = −0.66 e Å^−^^3^
96 parameters	Extinction correction: *SHELXL97* (Sheldrick, 2008), Fc^*^=kFc[1+0.001xFc^2^λ^3^/sin(2θ)]^-1/4^
3 restraints	Extinction coefficient: 0

### Special details

**Table:**

Geometry. All e.s.d.'s (except the e.s.d. in the dihedral angle between two l.s. planes) are estimated using the full covariance matrix. The cell e.s.d.'s are taken into account individually in the estimation of e.s.d.'s in distances, angles and torsion angles; correlations between e.s.d.'s in cell parameters are only used when they are defined by crystal symmetry. An approximate (isotropic) treatment of cell e.s.d.'s is used for estimating e.s.d.'s involving l.s. planes.
Refinement. Refinement of *F*^2^ against ALL reflections. The weighted *R*-factor *wR* and goodness of fit *S* are based on *F*^2^, conventional *R*-factors *R* are based on *F*, with *F* set to zero for negative *F*^2^. The threshold expression of *F*^2^ > σ(*F*^2^) is used only for calculating *R*-factors(gt) *etc*. and is not relevant to the choice of reflections for refinement. *R*-factors based on *F*^2^ are statistically about twice as large as those based on *F*, and *R*- factors based on ALL data will be even larger.

### Fractional atomic coordinates and isotropic or equivalent isotropic displacement parameters (Å^2^)

**Table:**

	*x*	*y*	*z*	*U*_iso_*/*U*_eq_	Occ. (<1)
M2	0.7500	0.04980 (16)	0.2500	0.0129 (3)	0.9400 (1)
M2MG	0.7500	0.04980 (16)	0.2500	0.0129 (3)	0.0600 (1)
M2(1)	0.7500	0.45601 (17)	0.7500	0.0169 (3)	
M1	0.7500	0.65310 (10)	0.2500	0.00495 (18)	0.8600 (1)
M1Fe	0.7500	0.65310 (10)	0.2500	0.00495 (18)	0.1400 (1)
M1(1)	0.7500	0.84745 (9)	0.7500	0.00855 (17)	0.8000 (1)
M11Fe	0.7500	0.84745 (9)	0.7500	0.00855 (17)	0.2000 (1)
Si1	0.04344 (7)	0.84714 (7)	0.22988 (13)	0.00748 (14)	
Si2	0.03811 (7)	0.66398 (7)	0.73666 (13)	0.00731 (14)	
O1	0.86242 (18)	0.8417 (2)	0.1106 (4)	0.0133 (4)	
O2	0.85803 (18)	0.6879 (2)	0.6524 (4)	0.0129 (4)	
O3	0.1201 (2)	0.0136 (2)	0.3071 (4)	0.0130 (4)	
O4	0.1012 (2)	0.4949 (2)	0.7949 (4)	0.0141 (4)	
O5	0.10966 (18)	0.7652 (2)	0.0132 (3)	0.0105 (3)	
O6	0.09535 (18)	0.74992 (19)	0.5087 (3)	0.0112 (3)	

### Atomic displacement parameters (Å^2^)

**Table:**

	*U*^11^	*U*^22^	*U*^33^	*U*^12^	*U*^13^	*U*^23^
M2	0.0162 (7)	0.0106 (7)	0.0093 (7)	0.000	0.0002 (5)	0.000
M2MG	0.0162 (7)	0.0106 (7)	0.0093 (7)	0.000	0.0002 (5)	0.000
M2(1)	0.0210 (7)	0.0107 (7)	0.0128 (8)	0.000	−0.0037 (6)	0.000
M1	0.0060 (4)	0.0045 (4)	0.0037 (4)	0.000	0.0006 (3)	0.000
M1Fe	0.0060 (4)	0.0045 (4)	0.0037 (4)	0.000	0.0006 (3)	0.000
M1(1)	0.0094 (3)	0.0085 (4)	0.0073 (4)	0.000	0.0020 (3)	0.000
M11Fe	0.0094 (3)	0.0085 (4)	0.0073 (4)	0.000	0.0020 (3)	0.000
Si1	0.0081 (3)	0.0074 (3)	0.0065 (3)	−0.0002 (2)	0.0016 (2)	−0.0005 (2)
Si2	0.0075 (3)	0.0076 (3)	0.0065 (3)	0.0000 (2)	0.0018 (2)	0.0004 (2)
O1	0.0103 (7)	0.0128 (9)	0.0165 (9)	0.0007 (6)	0.0039 (7)	−0.0023 (7)
O2	0.0074 (7)	0.0173 (9)	0.0132 (9)	−0.0013 (6)	0.0018 (6)	0.0035 (7)
O3	0.0200 (9)	0.0074 (8)	0.0106 (9)	−0.0014 (6)	0.0033 (7)	−0.0009 (6)
O4	0.0188 (9)	0.0104 (9)	0.0132 (9)	0.0006 (7)	0.0050 (7)	0.0002 (7)
O5	0.0098 (7)	0.0136 (9)	0.0078 (8)	0.0009 (6)	0.0024 (6)	−0.0018 (6)
O6	0.0125 (8)	0.0129 (9)	0.0086 (8)	−0.0017 (6)	0.0037 (6)	0.0017 (6)

### Geometric parameters (Å, °)

**Table:**

M2—O1^i^	2.319 (2)	M1—O2	2.0658 (18)
M2—O1^ii^	2.319 (2)	M1—O2^xi^	2.0658 (18)
M2—O3^iii^	2.3356 (18)	M1—O1^xi^	2.1917 (19)
M2—O3^iv^	2.3356 (18)	M1—O1	2.1917 (19)
M2—O6^v^	2.3651 (19)	M1(1)—O3^v^	1.8071 (19)
M2—O6^vi^	2.3651 (19)	M1(1)—O3^vii^	1.8071 (19)
M2—O5^vii^	2.7127 (19)	M1(1)—O1^xii^	1.8645 (18)
M2—O5^viii^	2.7127 (19)	M1(1)—O1^xi^	1.8645 (18)
M2(1)—O2^ix^	2.376 (2)	M1(1)—O2^ix^	1.8788 (19)
M2(1)—O2	2.376 (2)	M1(1)—O2	1.8788 (19)
M2(1)—O4^x^	2.4073 (19)	Si1—O3^xiii^	1.6058 (19)
M2(1)—O4^vi^	2.4073 (19)	Si1—O5	1.6216 (18)
M2(1)—O5^vii^	2.438 (2)	Si1—O6	1.6276 (18)
M2(1)—O5^v^	2.438 (2)	Si1—O1^xiv^	1.6294 (18)
M2(1)—O6^vii^	2.8944 (19)	Si2—O4	1.5725 (19)
M2(1)—O6^v^	2.8944 (19)	Si2—O2^xiv^	1.6326 (18)
M1—O4^vi^	1.9678 (19)	Si2—O6	1.6366 (18)
M1—O4^v^	1.9678 (19)	Si2—O5^xii^	1.6510 (18)
			
O4^vi^—M1—O4^v^	98.88 (11)	O3^v^—M1(1)—O2^ix^	170.80 (8)
O4^vi^—M1—O2	96.72 (7)	O3^vii^—M1(1)—O2^ix^	89.37 (8)
O4^v^—M1—O2	94.15 (7)	O1^xii^—M1(1)—O2^ix^	83.67 (8)
O4^vi^—M1—O2^xi^	94.15 (7)	O1^xi^—M1(1)—O2^ix^	94.09 (8)
O4^v^—M1—O2^xi^	96.72 (7)	O3^v^—M1(1)—O2	89.37 (8)
O2—M1—O2^xi^	163.26 (11)	O3^vii^—M1(1)—O2	170.80 (8)
O4^vi^—M1—O1^xi^	90.36 (7)	O1^xii^—M1(1)—O2	94.09 (8)
O4^v^—M1—O1^xi^	164.04 (7)	O1^xi^—M1(1)—O2	83.67 (8)
O2—M1—O1^xi^	71.74 (7)	O2^ix^—M1(1)—O2	85.52 (11)
O2^xi^—M1—O1^xi^	95.54 (7)	O3^xiii^—Si1—O5	109.12 (10)
O4^vi^—M1—O1	164.04 (7)	O3^xiii^—Si1—O6	104.39 (10)
O4^v^—M1—O1	90.36 (7)	O5—Si1—O6	109.41 (9)
O2—M1—O1	95.54 (7)	O3^xiii^—Si1—O1^xiv^	117.40 (10)
O2^xi^—M1—O1	71.74 (7)	O5—Si1—O1^xiv^	107.69 (9)
O1^xi^—M1—O1	83.83 (10)	O6—Si1—O1^xiv^	108.64 (10)
O3^v^—M1(1)—O3^vii^	96.67 (12)	O4—Si2—O2^xiv^	118.08 (10)
O3^v^—M1(1)—O1^xii^	89.08 (8)	O4—Si2—O6	111.93 (10)
O3^vii^—M1(1)—O1^xii^	92.94 (8)	O2^xiv^—Si2—O6	107.21 (9)
O3^v^—M1(1)—O1^xi^	92.94 (8)	O4—Si2—O5^xii^	107.11 (10)
O3^vii^—M1(1)—O1^xi^	89.08 (8)	O2^xiv^—Si2—O5^xii^	106.27 (9)
O1^xii^—M1(1)—O1^xi^	176.97 (12)	O6—Si2—O5^xii^	105.39 (9)

Symmetry codes: (i) −*x*+3/2, *y*−1, −*z*+1/2; (ii) *x*, *y*−1, *z*; (iii) *x*+1/2, −*y*, *z*−1/2; (iv) −*x*+1, −*y*, −*z*+1; (v) −*x*+1, −*y*+1, −*z*+1; (vi) *x*+1/2, −*y*+1, *z*−1/2; (vii) *x*+1/2, −*y*+1, *z*+1/2; (viii) −*x*+1, −*y*+1, −*z*; (ix) −*x*+3/2, *y*, −*z*+3/2; (x) −*x*+1, −*y*+1, −*z*+2; (xi) −*x*+3/2, *y*, −*z*+1/2; (xii) *x*, *y*, *z*+1; (xiii) *x*, *y*+1, *z*; (xiv) *x*−1, *y*, *z*.
